# A deep learning method for foot-type classification using plantar pressure images

**DOI:** 10.3389/fbioe.2023.1239246

**Published:** 2023-09-11

**Authors:** Yangyang Zhao, Jiali Zhou, Fei Qiu, Xuying Liao, Jianhua Jiang, Heqing Chen, Xiaomei Lin, Yiqun Hu, Jianquan He, Jian Chen

**Affiliations:** ^1^ School of Medicine, Xiamen University, Xiamen, Fujian, China; ^2^ Department of Rehabilitation, Zhongshan Hospital of Xiamen University, School of Medicine, Xiamen University, Xiamen, Fujian, China; ^3^ The School of Clinical Medicine, Fujian Medical University, Fuzhou, Fujian, China; ^4^ Department of Gastroenterology, Zhongshan Hospital of Xiamen University, School of Medicine, Xiamen University, Xiamen, Fujian, China

**Keywords:** flat feet, deep learning, ResNet-50, YOLO-v5, multilabel classification, foot type recognition

## Abstract

**Background:** Flat foot deformity is a prevalent and challenging condition often leading to various clinical complications. Accurate identification of abnormal foot types is essential for appropriate interventions.

**Method:** A dataset consisting of 1573 plantar pressure images from 125 individuals was collected. The performance of the You Only Look Once v5 (YOLO-v5) model, improved YOLO-v5 model, and multi-label classification model was evaluated for foot type identification using the collected images. A new dataset was also collected to verify and compare the models.

**Results:** The multi-label classification algorithm based on ResNet-50 outperformed other algorithms. The improved YOLO-v5 model with Squeeze-and-Excitation (SE), the improved YOLO-v5 model with Convolutional Block Attention Module (CBAM), and the multilabel classification model based on ResNet-50 achieved an accuracy of 0.652, 0.717, and 0.826, respectively, which is significantly higher than those obtained using the ordinary plantar-pressure system and the standard YOLO-v5 model.

**Conclusion:** These results indicate that the proposed DL-based multilabel classification model based on ResNet-50 is superior in flat foot type detection and can be used to evaluate the clinical rehabilitation status of patients with abnormal foot types and various foot pathologies when more data on patients with various diseases are available for training.

## Introduction

The feet comprise bones, muscles, and ligaments and enable seamless movement and direct contact with the ground during walking. However, congenital foot shape problems or walking with an uncomfortable gait, such as in-toeing and out-toeing gait, can result in concentrated ground pressure on specific parts of the foot, resulting in permanent deformation of the foot and may cause knee joint or back pain (Arunakul et al., 2013; Izraelski, 2013). The feet may get easily deformed due to wrong walking postures; moreover, foot deformations not only pose a threat to foot health but also cause fatigue and pain while walking and can even result in spine deformation. Therefore, accurate diagnosis of foot deformations is crucial.

Flatfoot is a common orthopedic condition characterized by the collapse of the medial longitudinal arch (MLA) and is often accompanied by calcaneal valgus and talonavicular joint abduction. It may cause plantar pain or fatigue after exercise (Arunakul et al., 2013; Carr et al., 2016). In children, developmental flat foot can be caused by many factors and may be symptomatic or asymptomatic and flexible or rigid. For example, the cause may be abnormal bone and joint development, such as with a tarsal coalition, a congenital vertical talus, or an accessory navicular bone. The soft tissue of generalized ligamentous laxity from Marfan’s or Ehlers–Danlos can also result in flat foot deformity (McCormack et al., 2001; Flores et al., 2019). Adult flat foot can be categorized as either residual flat foot deformity from a developmental cause or as an acquired flat foot. Acquired flat foot is associated with a tight triceps surae or isolated gastrocnemius tightness, posterior tibial tendon dysfunction, midfoot laxity, abduction of the forefoot, external rotation of the hindfoot, subluxation of the talus, traumatic deformities, ruptured plantar fascia, Charcot’s foot, and neuromuscular imbalance (polio, cerebral palsy, closed head injury, or following a cerebrovascular accident) (McCormack et al., 2001; O’Leary et al., 2013).

Flat foot deformity is often overlooked due to difficulties in accurate diagnosis, often leading to severe consequences. Attempts have been made to diagnose flat foot deformity by using the footprint index, MLA, and arch height index (Adoración Villarroya et al., 2008; Chang et al., 2014; Drefus et al., 2017). Nikolaidou and Boudolos, (2006) proposed a classification method based on static footprints to distinguish different foot types; however, these classifications are subjective, imprecise, and cannot quantify the changes before and after treatment. The 3D measurement system based on the footprint index has drawbacks, including variations caused by observer bias, equipment costs, and instrument calibration disparities. Although two-dimensional plantar image detection is user-friendly, it has low sensitivity (Su et al., 2017).

Foot deformity detection involves a classification process; therefore, improving the accuracy of classification is crucial. Deep learning (DL) methods are machine learning (ML) techniques that enable the computer to learn from data by extracting features from the data without human intervention, which is beneficial for professional data analysis applications (LeCun et al., 2015). Deep neural networks can extract features and identify and locate targets through backpropagation and parameter tuning (Deng et al., 2020; Lin et al., 2020). Region-based convolutional neural network (R-CNN) is a typical DL target recognition model and employs a multiscale feature pyramid and sliding window method for region proposal and bounding box detection. This two-stage pipeline greatly improves recognition accuracy (Redmon et al., 2016). You Only Look Once (YOLO) is a novel DL target detection model that uses anchors to concurrently localize and classify targets during convolutional feature extraction. Anchors leverage prior knowledge to design multiscale fixed reference boxes covering all positions and scales in an image. Each anchor predicts targets by using intersection over union (IoU) as a measure of detection accuracy; if the IoU exceeds a threshold, the number of learnable parameters reduces, and the efficiency improves (Albahli et al., 2020). Region-based convolutional neural network (R-CNN) and YOLO have been used in various fields; for example, in real-time vehicle recognition, R-CNN identifies and locates vehicles separately in an image, whereas YOLO performs one-stage target detection, concurrently localizing, classifying, and detecting vehicles. Although static footprint images can be used to detect foot morphology and pressure patterns (Yu et al., 2019), existing image classification methods rely on individual features and lack consistency, resulting in reduced precision and sensitivity. Therefore, we proposed a DL model for predicting foot type based on pressure distribution of the foot to more accurately and efficiently identify flat foot deformity and provide guidance for clinical diagnosis, treatment, and prognosis.

## Material and methods

### Data preparation

**FIGURE 1:**
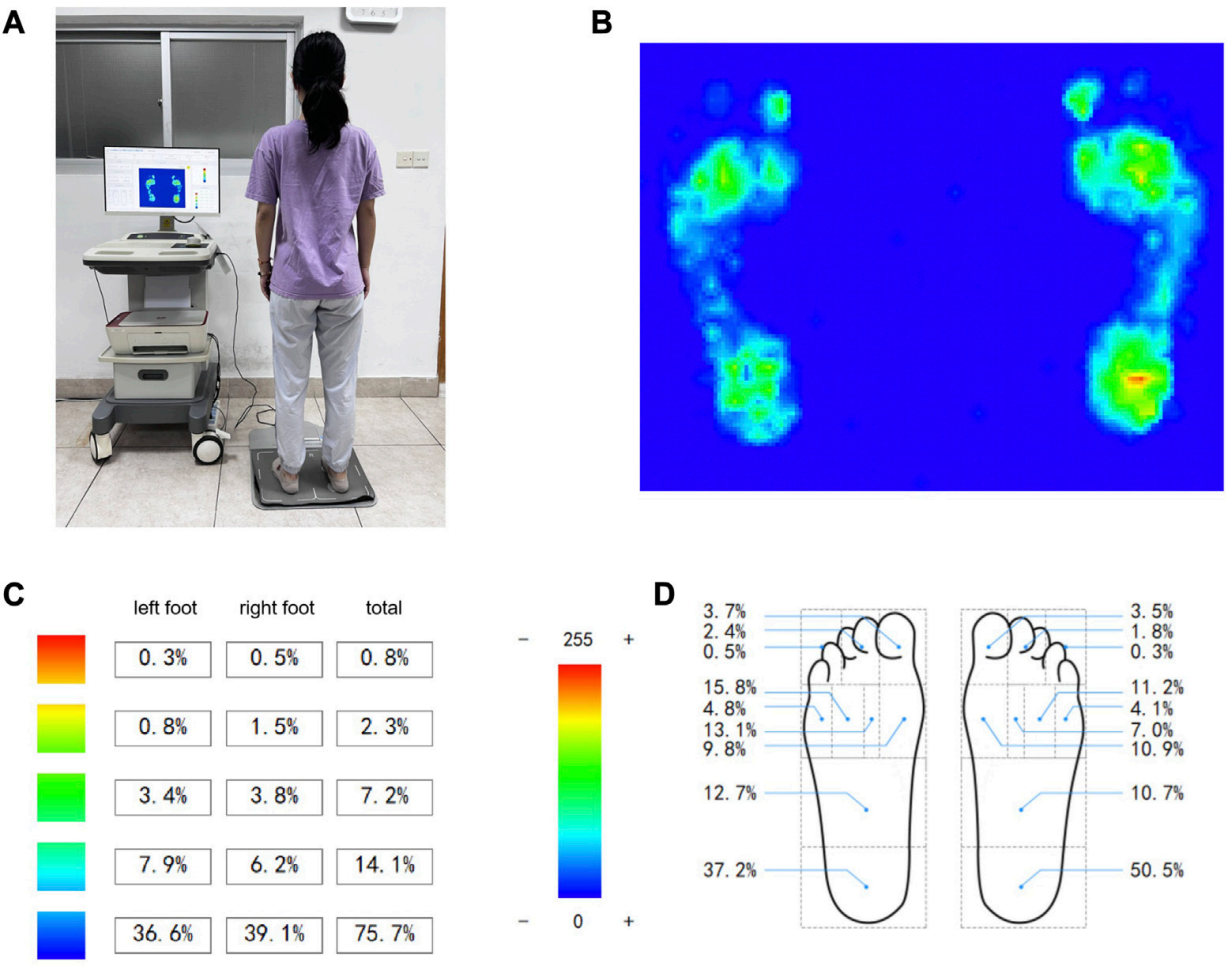
Collection of subjects’ plantar pressure and proportional distribution. **(A)** FeetMapping^Ⓡ^ Plantar Pressure Plate system. **(B)** Plantar pressure image. **(C)** Image pressure distribution ratio. **(D)** Proportional distribution of pressure in each area of plantar.

We retrospectively collected plantar pressure images and clinical data from flat foot patients and healthy volunteers during June 2021–June 2022. In case two physiotherapists disagreed on the diagnosis, a third researcher (Chen J) was consulted. The FeetMapping^Ⓡ^ Plantar Pressure Plate system (NeuCognic, Jiangsu, China) was used to obtain the plantar pressure images (Yu et al., 2019). This system comprises a pressure board with an effective area of 0.11 m^2^, a power interface, a network interface, a power switch, and an indicator light. During the test, subjects stood naturally for 30 s, and the tester collected raw data based on the plantar pressure distribution and stored it on a computer (Figure 1). An arch score of 20%–26% was considered normal. This research was conducted in accordance with the Declaration of Helsinki and was approved by the Ethics Committee of Zhongshan Hospital, which is affiliated to Xiamen University.

### Diagnostic criteria

MLA collapse, physical examination, and X-ray examination were used to diagnose flat foot (Meehan and Brage, 2003). The midpoint of the calcaneus, the midpoint of the inner and outer malleolus, and the midpoint of the lower third of the calf were traced and connected. The normal range of the Angle obtained was −5° to +5°, and the angle less than −5° was the deformity of foot varus (Flores et al., 2019). Moreover, A drop of 10 mm or more of the navicular bone will be interpreted as a flat foot (Persiane et al., 2021). Therefore, further X-rays are taken. The X-ray film is made from the lowest point of the calcaneus to the lowest point of the talar bone in a straight line, and then from the lowest point of the talar bone to the lowest point of the first metatarsal head in a straight line, the normal range of the Angle between the two lines is 113°–130°, more than 130° will be diagnosed as flat foot (Ueki et al., 2019; Caravaggi et al., 2021).

### Inclusion and exclusion criteria

Inclusion criteria: 1) age range: 5–60 years; 2) patients with a normal gait and no motor system diseases; 3) no history of foot trauma or operation; 4) all arches were determined by X-ray examination.

Exclusion criteria: 1) obvious foot deformity; 2) presence of high arches; 3) a history of lower limb and foot fractures or ankle sprain resulting in ligament damage or articular cartilage damage; 4) leg length discrepancy >2 cm (Vogt et al., 2020); 5) patients with cognitive dysfunction who are unable to cooperate with the test; 6) missing data.

For healthy subjects, the inclusion and exclusion criteria were consistent, except for not meeting the diagnostic criteria for flat feet.

### Image dataset processing and classification

**FIGURE 2:**
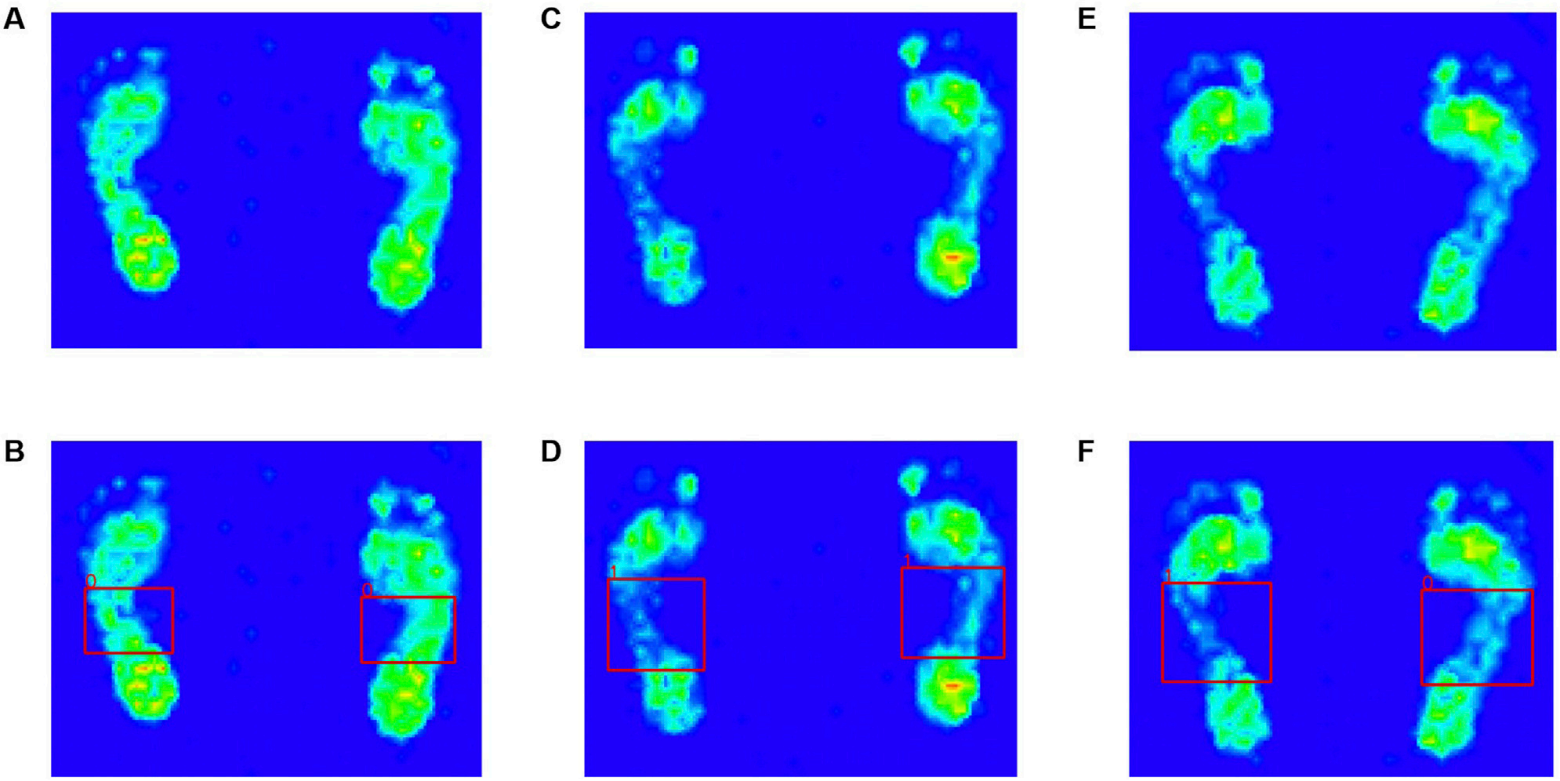
Visual plantar pressure image obtained by plantar pressure plate system. **(A)** flat feet, **(C)** healthy feet, **(E)** patients with one flat foot. The red dashed boxes refer to the ground truth. The three images below are visualized results of manual labeling by the physician using labelImg. **(B)** flat feet, **(D)** healthy feet, **(F)** patients with one flat foot.

After anonymizing the collected plantar pressure images, two professionally trained rehabilitation therapists labeled them; labelImg(1.8.6) was used to select the midfoot of the images for labeling classification (Figure 2). A text file containing the category, target center coordinates, target boundary frame, and aspect ratio information of the overall image corresponding to the original image was generated. Images were labeled as flat foot (class 0) or healthy foot (class 1), denoted by values of 0 and 1, respectively. The four corners of the middle foot region were selected as the center points in each image and recorded in the txt file by using (*x*,*y*) coordinates. A random seed 0) was used to divide the total dataset into a training set and a test set in the ratio of 8:2.

Target detection algorithms can be classified as two-stage detectors with region proposal networks and one-stage detectors. Although two-stage detectors yield high accuracy, their size makes deployment on embedded devices difficult. To improve abnormal foot-type screening using plantar pressure systems, we explored the use of YOLO, a one-stage detector, for its performance and efficiency. The YOLOv5 model has four variants, namely, YOLOv5s, YOLOv5 m, YOLOv5 l, and YOLOv5x; among these, YOLOv5s has a relatively small model size and is thus suitable for embedded applications. The model architecture can be divided into three parts: CSPDarkNet53 for feature extraction (Bochkovskiy et al., 2020), FPN module and PAN module for feature fusion and transmission, and the category and position prediction module. By utilizing YOLOv5s, we aimed to achieve real-time foot type detection on low-power devices. During inference, when there is a target in the specified cell, the IoU between the bounding box and the true target can be calculated using Eq. 1:

$${\text{IoU}}_{\text{pred}}^{\text{truth}}=\frac{\text{ground}\;\text{truth}\;\text{box}\cap \text{predicted}\;\text{bounding}\;\text{box}}{\text{ground}\;\text{truth}\;\text{box}\cup \text{predicted}\;\text{bounding}\;\text{box}}$$ (1)

Predicted category information and target box confidence are multiplied to obtain the category confidence score for each target box (Eq. 2):

$$\text{Class specific confidence score}={\mathrm{Pr}}_{\left({\text{class}}_{i}|\text{object}\right)}\times {\mathrm{Pr}}_{\left(\text{object}\right)}\times {\text{IOU}}_{\text{pred}}^{\text{truth}}.$$ (2)

However, YOLOv5s has limited learning capacity. Attention mechanisms enhance object detection models by improving their capability to learn representations. Attention modules require very few additional parameters, thereby enhancing capabilities without substantially increasing model complexity.

### Improved YOLOv5s algorithm

Squeeze-and-Excitation Networks (SENet) (Hu et al., 2018) and Convolutional Block Attention Module (CBAM) (Woo et al., 2018) are commonly used attention mechanism modules. SENet improves feature learning by modeling channel relationships, whereas CBAM focuses on informative regions and suppresses irrelevant regions by sequentially inferring spatial and channel attention. The simple and efficient designs of SE, CBAM, and other attention modules enable easy integration into different layers of YOLOv5s, thereby yielding considerable performance gains. Incorporating attention mechanisms into the C3 module (the third residual block of the CSPDarknet53 backbone network) enhances feature representation. Therefore, in this study, we incorporated SE, CBAM, and other attention modules into the C3 module. The SE module involves squeeze and excitation operations. The squeeze operation performs global average pooling to reduce the output feature map of the C3 block with size H × W × C to a feature map *Z* of size 1 × 1 × C (Eq. 3):

$$Z=\frac{1}{H\times W}\sum_{i=1}^{H}\sum_{j=1}^{W}u_{C}\left(i,j\right)$$ (3)

The excitation operation learns a channel weight vector *S*. σ denotes the sigmoid activation function, *W*_*1*_ and *W*_*2*_ are learnable parameter matrices, and δ denotes the ReLU activation function for channel recalibration. Channel recalibration is performed using the excitation operation (Eq. 4):

$$S=\sigma \left(W_{1}\delta W_{2}\left(Z\right)\right)$$ (4)

After processing using the SE module, channel attention improves important features while suppressing irrelevant ones.

The CBAM module contains channel attention and spatial attention branches. We denoted the output feature map of the C3 block as *U* with size H × W × C. The channel attention branch comprises global max pooling and average pooling operations, which transform U into two one-dimensional descriptor vectors. These vectors are processed by respective multilayer perceptrons and combined. Next, a sigmoid activation generates channel weights from 0 to 1. Finally, the learned channel weight coefficients are multiplied by the input feature map $u_{X}$ to obtain the channel-wise attended output feature map $Z_{C}$ (Eq. 5):

$$Z_{C}=\sigma \left({MLP\left(F\right.}_{\max}\left(u_{X}\right)\right)+{MLP\left(F\right.}_{avg}\left(u_{X}\right)\left.)\right)\times u_{X}$$ (5)

First, the spatial attention branch compresses the input $u_{X}$ along the channel dimension by applying global max pooling and average pooling across the channels to generate two 2D feature maps. Next, these feature maps are concatenated channel-wise to form a tensor with two channels, which is further convolved using a 7 × 7 kernel to reduce to a single channel. A sigmoid activation then generates spatial attention coefficients. Finally, the input $u_{X}$ is multiplied by these coefficients to obtain the spatially attended output feature map $Z_{X}$ (Eq. 6):

$$Z_{X}=\sigma \left({{{Conv}_{7\times 7}\left(F\right.}_{\max}}^{\prime }\left(u_{X}\right)\right){{{,Conv}_{7\times 7}\left(F\right.}_{avg}}^{\prime }\left(u_{X}\right)\left.)\right)\times u_{X}$$ (6)

**FIGURE 3:**
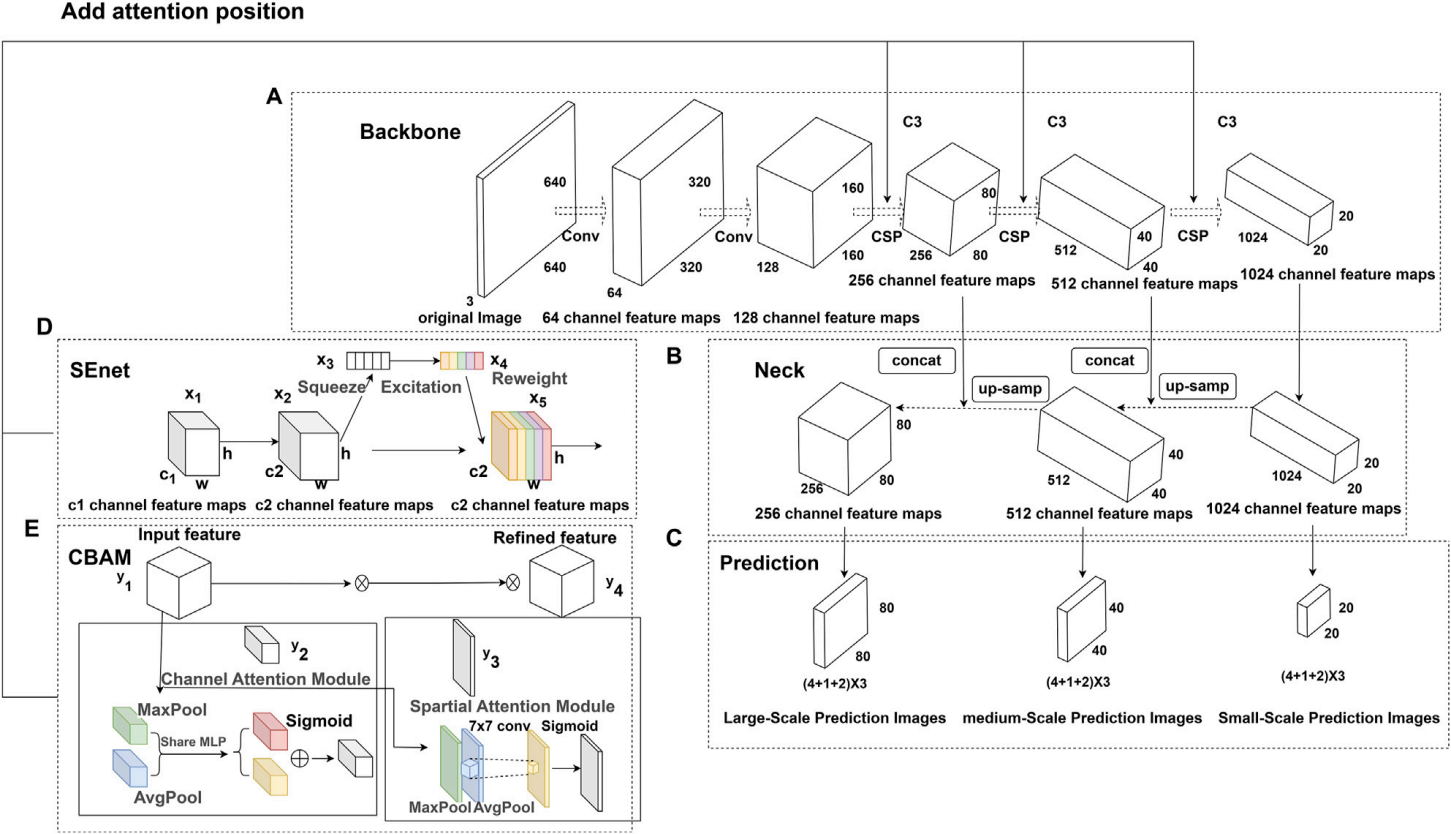
YOLO-v5 and improved YOLO-v5 base modules. **(A)** YOLO-v5 core network. It is composed of two 6 × 6 convolutions and three CSPDarkNet53 network modules. For each CSP module, the feature graph size is reduced to half of the original size, and the feature channel is doubled. **(B)** The Neck part of YOLO-v5. Using the classic structure of feature pyramid, from top to bottom, all scales are connected and fused to construct a high-level semantic feature map. **(C)** Multiscale prediction head for YOLO-v5. It predicts three kinds of prediction charts with different aspect ratios. Each scale prediction picture will predict three kinds of target anchor frames at the same time. The target anchor frame needs to predict four positions, confidence and classification information of anchor frames. **(D)** SE attention structure. Through squeeze global pooling operation, the spatial dimensions can be compressed into feature graphs with the size of 1 × 1×C, and global and weight information can be extracted. **(E)** CBAM attention structure. It can not only learn the importance of each feature channel independently, but also add the maximum scaling operation which can obtain important information about each feature channel and feature space at the same time.

Two attention mechanisms are added to the CSPDarkNet53 module, as shown in Figure 3.

### Other strategies

For data augmentation, we randomly cropped the training set to images of size 640 × 640 × 3. The sizes of nine prior boxes were obtained through K-means clustering: 10 × 13, 16 × 30, 33 × 23, 30 × 61, 62 × 45, 59 × 119, 116 × 90, 156 × 198, and 373 × 326. For training, we divided the feature map into grids of the same size as the prior box. When the target center was within a grid, that specific grid unit was responsible for target detection. It outputted a prediction box based on the initial anchor box and then compared it with the real box, calculated the discrepancy between the two, and then iteratively updated the network parameters.

**FIGURE 4:**
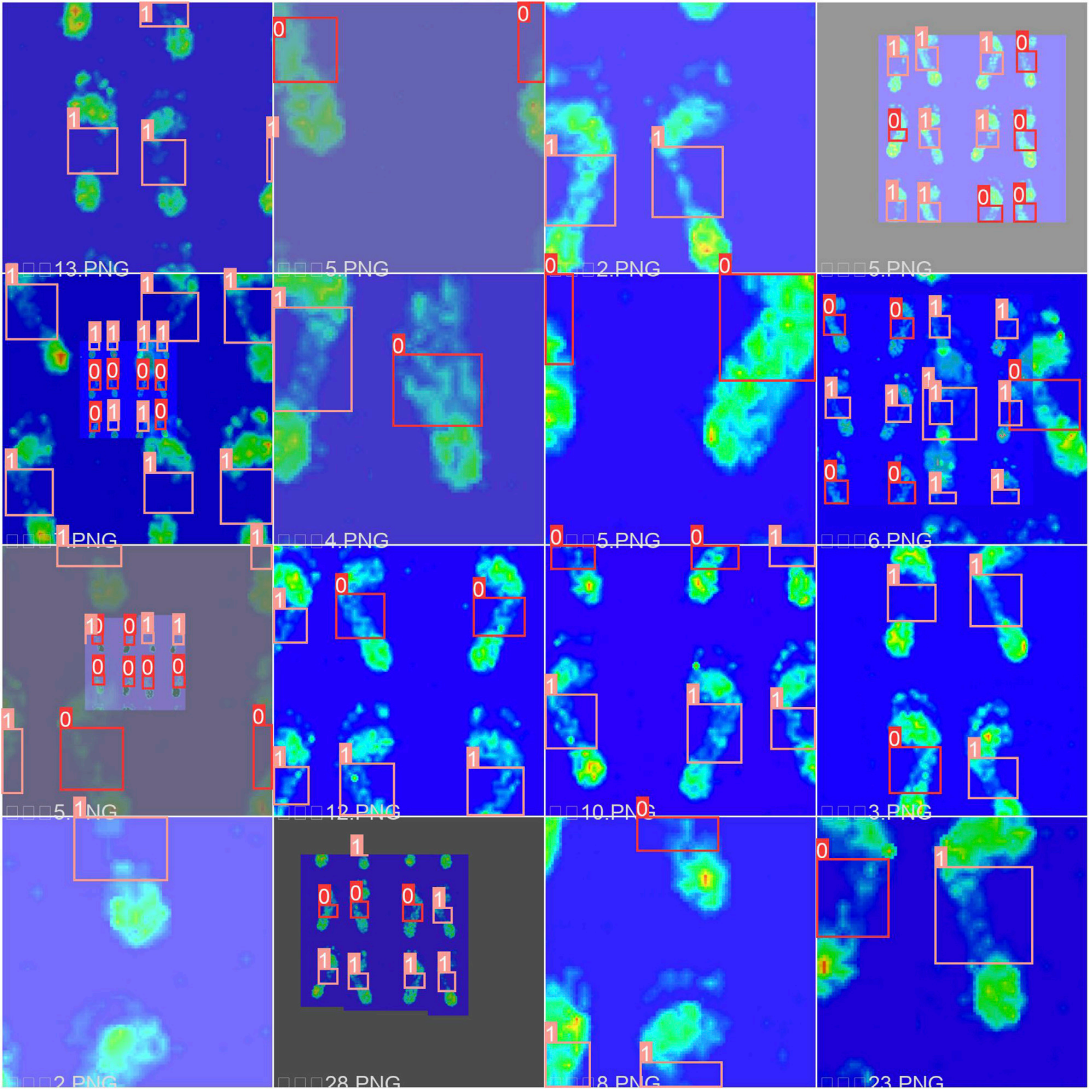
The results of the data enhancement. We randomly alter the hue, saturation and value of images by 15%, 70% and 40% respectively. Horizontal with 50% probability mosaic augmentation with 100% probability and mixup augmentation with 10% probability are used to images.

In this study, we improved the generalizability by using enhancement techniques. Previous studies have mainly utilized random rotation, scaling, panning, flipping, and illumination to augment data. However, such techniques have limitations, such as poor generalization, information loss, and noise interference. Therefore, to maximize algorithm performance, we adopted mosaic data augmentation following YOLOv4 and YOLOX (Zheng et al., 2022), random affine transformation, mixup augmentation (Zhang et al., 2018), and HSV enhancement (Redmon et al., 2016). Experimental results demonstrated that these enhancement methods improved detection accuracy and enhanced model generalization in the YOLOV5 framework. The augmentation results are shown in Figure 4.

### Multilabel classification

**FIGURE 5:**
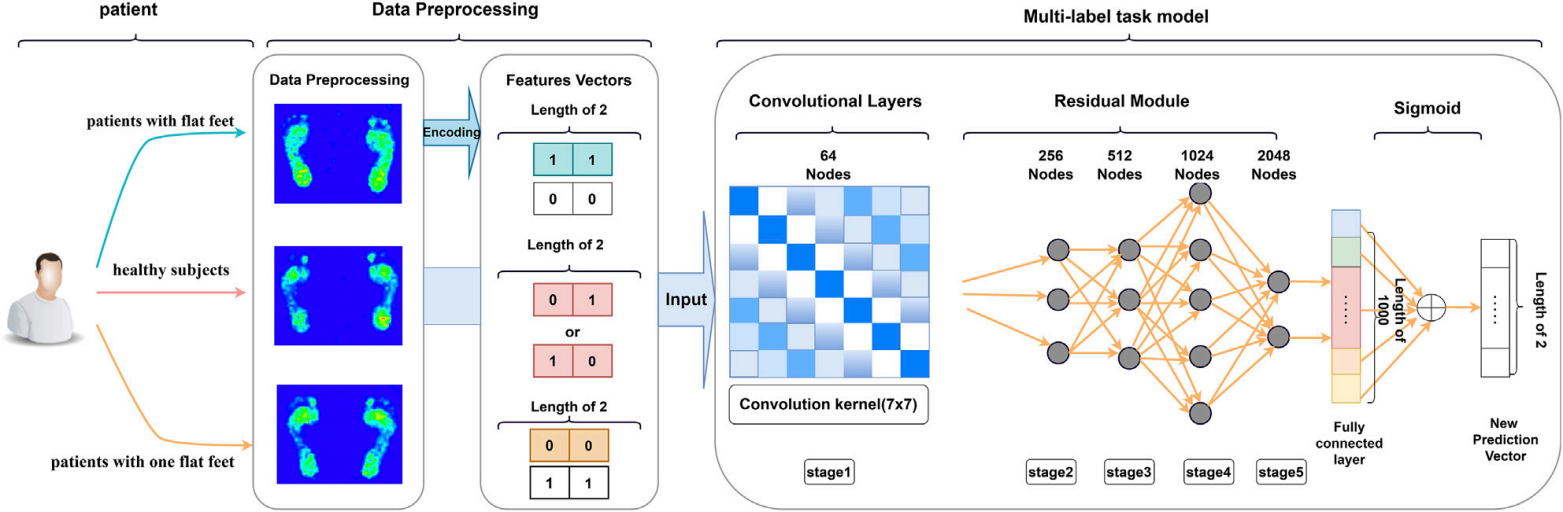
Pipeline of the proposed method for multilabel classification task.

Studies have demonstrated the importance of the midfoot region in flatfoot recognition (Welte et al., 2023). However, Buldt et al. (2018) studied the difference in the ratio of plantar pressure distribution between normal and flat feet and found that the ratio of plantar pressure distribution between the two foot types differs in different regions. Therefore, to determine whether these additional regions aid flatfoot identification, we employed a multilabel classification model to extract features and categorize plantar pressure images. We used ResNet-50 in the backbone network because it is a dynamic convolution neural network architecture with good performance. We resized the image size of the dataset to 256 × 256 pixels, assigned two labels to the single original image, namely flat foot and healthy foot, and then performed one-hot encoding. Healthy feet were assigned the code (0,0), whereas flat feet were assigned (1,1). In cases where only one arch collapsed, the coding label was set as 0, 1 (or 1, 0), and training was conducted for a total of 100 epochs, as depicted in Figure 5.

### Statistical analysis

We used SPSS 26.0 for the statistical analysis of participants’ general information and expressed the measurement data as Mean ± Standard Deviation. In addition, we used independent sample’s *t*-test to verify the differences between groups, with statistically significance set at *p* < 0.05.

## Results

The inclusion criteria yielded 125 subjects: 61 with normal feet, 61 with flat feet, and 3 with single flat foot. Each person underwent the tests 13 times. A total of 1,573 plantar pressure images were obtained: 748 flat feet, 51 single flat foot, and 774 normal feet. Basic information is presented in Table 1, revealing no statistical difference between the two groups (*p* > 0.05).

**TABLE 1:** General information of subjects.

	Normal foot (*n* = 61)	Flat foot (*n* = 64)	*P*
Female, n	37	37	
Age, y	32.64 ± 14.85	29.56 ± 17.09	0.286
Body mass (kg)	55.83 ± 12.94	54.00 ± 17.44	0.508
Body mass index (kg/m^2^)	20.92 ± 2.86	21.72 ± 4.40	0.231

### Experimental environment

The laboratory setup comprised Ubuntu 20.04.1 LTS, an Intel Xeon E5–2,620 v4 2.10 GHz processor, and four NVIDIA TITAN Xp-12 GB GPUs. Python 3.7.5 was the development environment, and PyTorch 1.12.1 was the DL framework, and CUDA 11.3/CUDNN 8.2 was used for image processing.

### Evaluation indicators

We used accuracy, precision, recall, F1-score, average precision (AP), and mean average precision (mAP) to evaluate the merits of models (Eq. 7):

$$\text{Accuracy}=\frac{\text{TP}+\text{TN}}{\text{TP}+\text{FN}+\text{FP}+\text{TN}}$$

$$\text{Precision}=\frac{\text{TP}}{\text{TP}+\text{FP}}$$

$$\text{Recall}=\frac{\text{TP}}{\text{TP}+\text{FN}}$$

$$F1-\text{score}=2\times \frac{\text{Precision}\times \text{Recall}}{\text{Precision}+\text{Recall}}$$

$$\text{AP}=\int_{0}^{1}\text{Precision}\left(\text{Recall}\right)d\text{Recall}$$

$$\text{mAP}=\frac{1}{C}\sum c\epsilon C\int \text{AP}\left(c\right)$$ (7)

True positives (TPs) are correctly classified positive samples, false positives (FPs) are incorrectly classified positives, and false negatives (FNs) are incorrectly classified negatives. Accuracy is the proportion of correctly classified samples. Precision is the ratio of TPs to total predicted positives. Recall is the ratio of TPs to total actual positives. F1-score is the harmonic mean of precision and recall. AP and mAP are commonly used in target detection to assess a model’s detection effectiveness and performance (Zhao et al., 2022). AP integrates the precision *P* at each discrete recall *R* point from 0 to 1 by calculating the area under the precision-recall curve. A higher AP value indicates better detection performance for a certain class. Let *C* denote the total number of classes in the detection model, *c* denote each class, and AP(c) denote the AP for each class *c*. mAP is computed by first calculating the AP for each class and then averaging over the AP across all classes, resulting in a comprehensive evaluation metric that reflects the model’s detection effectiveness for all categories.

## Model training

### Training of attention-enhanced YOLOv5s

The following steps are performed to train the model. To obtain better training results, we initialized the YOLOv5s model by using weights pretrained on the COCO train 2017 dataset and optimally trained the model by using adaptive weight (AdaW) (Loshchilov and Hutter, 2019). Next, we resized the input images to 640 × 640 pixels. We employed AdamW for parameter optimization and adopted a maximum learning rate of 0.1, with a batch size of 16 images per iteration. The first three epochs employed frozen training, where the weights of the feature extraction layers were maintained constant. This was followed by 97 epochs of unfrozen training for fine-tuning all layers in the model. The IoU threshold and momentum were set as 0.2 and 0.937, respectively. We used the same training method for the other versions of YOLOv5s in this study.

### Training of multilabel classification

To fit the dataset, we adopted a fine-tuning approach by modifying the last fully-connected layer of ResNet-50. We loaded the model on the GPU for training by using binary cross-entropy loss. Furthermore, we utilized the stochastic gradient descent optimizer with differentiated learning rates, where the newly added classifier layers assume a 10× larger rate of 0.001 for rapid adaptation. The learning rates were reduced by 10× every 5 epochs by using step decay scheduling. The model was trained for 100 epochs to obtain an adapted network suited for the specific dataset.

### Plantar pressure

Compared with normal subjects, subjects with flat feet exhibited lower pressure in the forefoot (toe and metatarsal region) and higher pressure in the middle foot (*p* < 0.05); in addition, no significant difference was observed in the heel pressure ratio between the two groups (*p* > 0.05) (Table 2).

**TABLE 2:** Ratio of pressure distribution in the plantar area between the two groups (%).

	Normal foot (*n* = 61)	Flat foot (*n* = 64)	*P*
Left toe	6.49 ± 3.06	4.53 ± 1.73	<0.001
Left metatarsal	42.64 ± 6.41	38.13 ± 9.74	0.003
Left mediopodium	12.97 ± 6.69	21.56 ± 7.68	<0.001
Left heel	37.91 ± 7.60	35.79 ± 8.59	0.147
Right toe	6.84 ± 3.00	4.95 ± 2.10	<0.001
Right metatarsal	41.82 ± 7.30	36.97 ± 9.66	0.002
Right mediopodium	13.15 ± 6.02	21.99 ± 7.12	<0.001
Right heel	38.17 ± 9.46	36.11 ± 8.71	0.205

## Model performance evaluation

### Comparison between the deep learning model and traditional machine learning model

To verify the test performance of the model and the FeetMapping^Ⓡ^ plantar pressure measuring system (NeuCognic, Jiangsu, China), we inputted the test dataset into the improved trained YOLO-v5 model and the FeetMapping^Ⓡ^ plantar pressure measuring system (NeuCognic, Jiangsu, China) for statistical verification. The prediction accuracy of YOLOv5 model based on the attention mechanism was higher than that of traditional ML in both healthy and flat foot patients (Table 3).

**TABLE 3:** Accuracy of deep learning models and traditional machine learning models (%).

	YOLO-v5 based on attention mechanism	FeetMapping^Ⓡ^machine
All	84.7	76.5
Patient	86.5	76.0
Health	82.9	75.8

### Performance comparison between improved YOLO-v5 and original YOLO-v5

**FIGURE 6:**
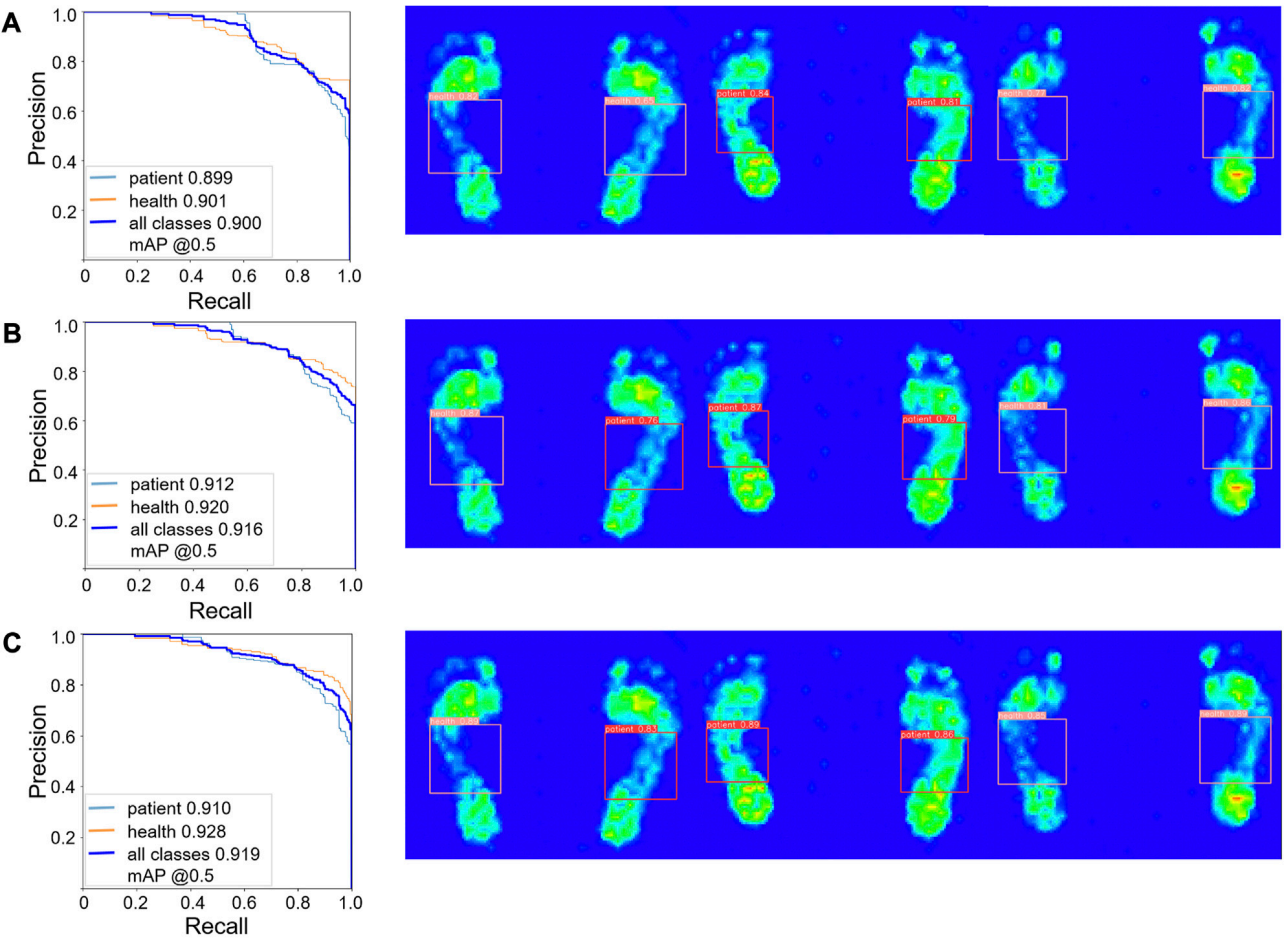
PR curves of three different models. **(A)** Ordinary YOLO-v5 model. **(B)** YOLO-v5 model with SE attention mechanism added. **(C)** YOLO-v5 model with the CBAM attention mechanism added.

The improved YOLO-v5 model included SE and CBAM attention mechanisms in the C3 module. We compared the performance of the original YOLO-v5 model with the improved YOLO-v5 model by using precision-recall curve (PR curve). PR curve is commonly used when the distribution of data categories is uneven. The horizontal axis represents recall, and the vertical axis represents accuracy. Similar to the ROC curve, when the PR curve is closer to the upper right, it indicates that the model performance is better. The PR curve of YOLO-v5 model based on C3CBAM attention mechanism exhibited the best performance (Figure 6). The average accuracy and sensitivity of YOLO-v5 based on the CBAM attention mechanism were 84.7%, 86.4%, respectively, and the mAP for different IoU thresholds (0.5, 0.55, 0.6, 0.65, 0.7, 0.75, 0.8, 0.85, 0.9, and 0.95) was 91.9%. The average accuracy was 8.5% higher than that of YOLO-v5 with SE and 4.7% higher than that of YOLO-v5 without attention mechanism. Furthermore, the sensitivity was 5.4% lower than that of YOLO-v5 with SE and 2% lower than that of YOLO-v5. The average mAP for different IoU thresholds was 0.3% higher than that of YOLO-v5 with SE and 1.9% higher than that of YOLO-v5.

### Performance evaluation of multilabel classification tasks

**FIGURE 7:**
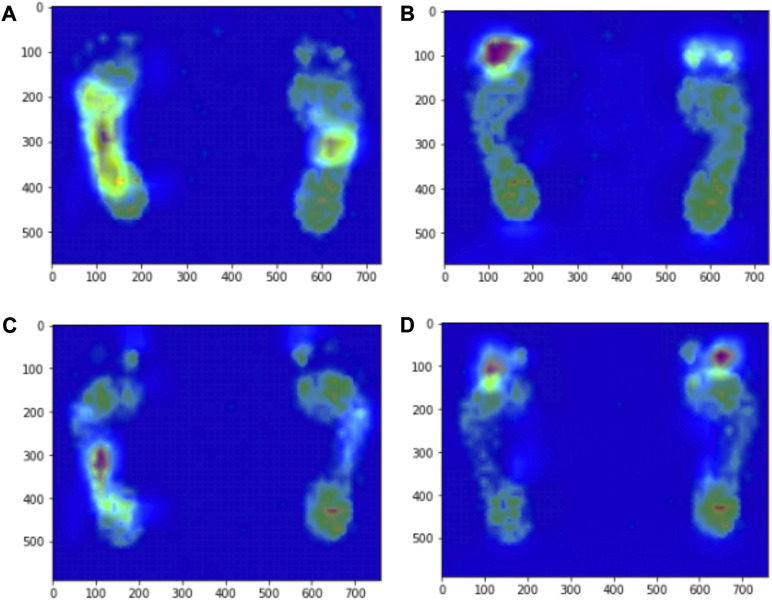
Grad-CAM heat map based on ResNet-50 model. ResNet-50 model sets the recognition object to normal foot, **(A)** the high activation region of pixel sensitivity of visual prediction images is located in the middle foot region, **(B)** the high activation area of pixel sensitivity of visual prediction images is located in the metatarsal region. ResNet-50 model sets the recognition object as flat feet, **(C)** the high activation region of pixel sensitivity of visual prediction images is located in the middle foot region, **(D)** the high activation area of pixel sensitivity of visual prediction images is located in the metatarsal region.

The distribution ratio of the participants’ plantar pressure regions revealed that the target detection task focused only on extracting features from the middle foot and overlooked the features of the toe, metatarsal bone, and hindfoot due to the specific labels assigned. To investigate the effect of plantar pressure on foot type detection capability, we generated thermal maps by using Grad-CAM in a multilabel classification task based on ResNet-50 (Figure 7). Thermal maps enable backpropagation through category assignment, obtaining gradient information from extracted features, and weighting the elements that significantly contribute to algorithmic recognition. When the recognition object was set as a normal foot, the highlighted regions were concentrated in the metatarsal and middle foot regions. Similarly, when the recognition object was set as a flat foot, the highlighted regions were primarily concentrated in the metatarsal and middle foot areas.

To further assess the accuracy of the multilabel sorting task, we randomly selected 20% of the dataset as the test set and used the remaining 80% for training. We employed different DL models for foot-type recognition of plantar pressure images based on the label features extracted in this study. We compared the performance of four models, namely, YOLO-v5, improved YOLO-v5 incorporating different attention mechanisms (SE and CBAM), and multilabel classification task based on ResNet-50. The results showed that although the accuracy, recall rate, and F1-score of the algorithm improved after adding attention mechanisms in YOLO-v5, the performance of the multilabel classification algorithm based on ResNet-50 was significantly superior to other algorithms (Table 4).

**TABLE 4:** Precision comparison of deep learning models.

Model	Precision (%)	Recall (%)	F1-score (%)
YOLO-v5	77.6	83.8	80.6
YOLO-v5_C3SE	76.2	91.8	83.3
YOLO-v5_C3CBAM	84.7	86.4	85.6
Multilabel_ResNet-50	91.8	92.9	92.3

### Model verification

**FIGURE 8:**
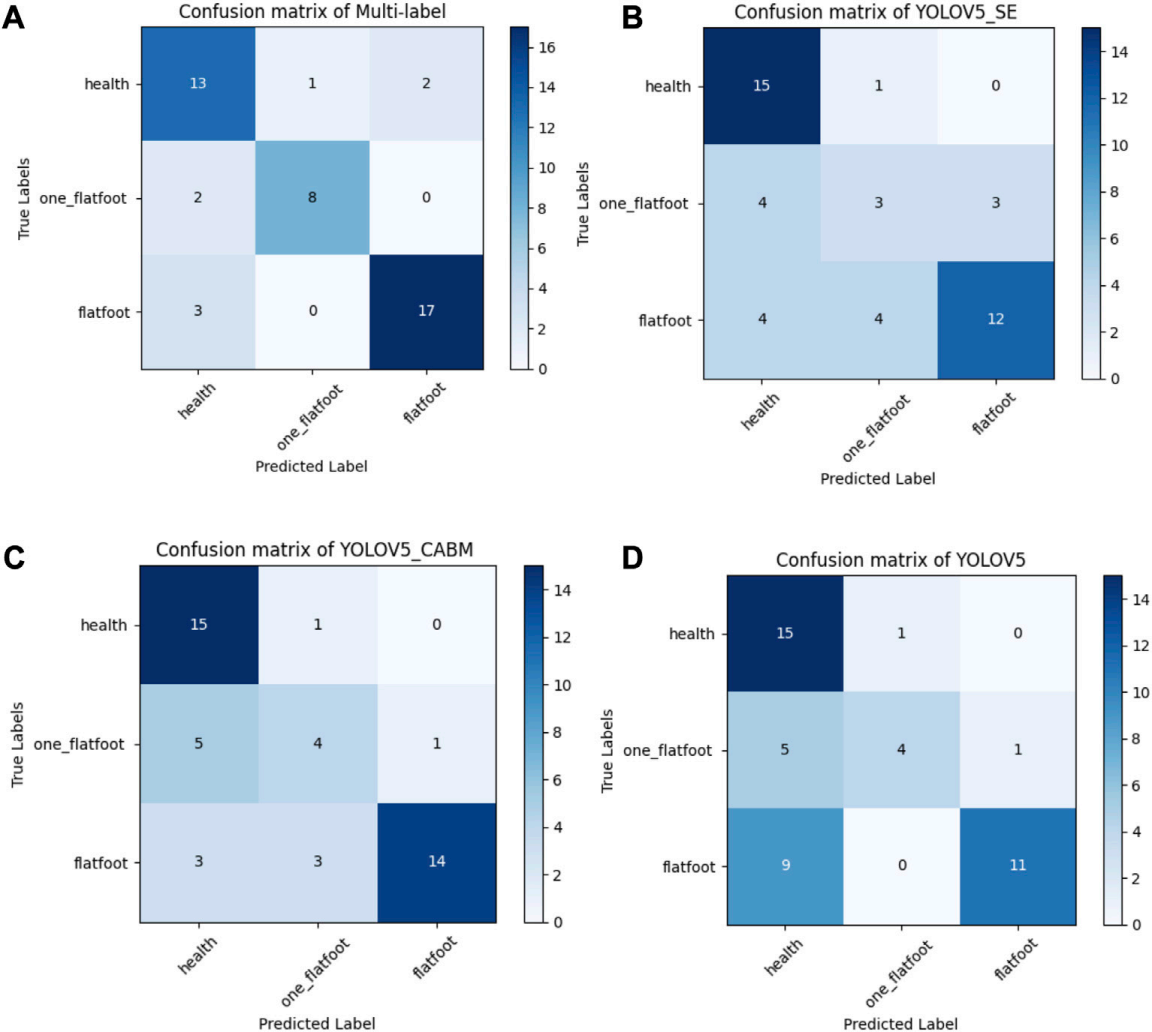
The confusion matrix of four different models. **(A)** For the confusion matrix of multilabel task model, the sensitivity and specificity of flatfoot recognition were 89.5% and 83.3%, respectively, and the specificity of one-foot flat recognition was 97.2%. **(B)** For the confusion matrix of YOLO-v5_SE, the sensitivity and specificity of flatfoot recognition were 100% and 68%, respectively, and the specificity of one-foot flat recognition was 96.9%. **(C)** For the confusion matrix of YOLO-v5_CBAM, the sensitivity and specificity of flatfoot recognition were 100% and 73.9%, respectively, and the specificity of one-foot flat recognition was 97%. **(D)** For the confusion matrix of YOLO-v5, the sensitivity and specificity of flatfoot recognition were 100% and 65.4%, respectively, and the specificity of one-foot flat recognition was 97.2%.

To validate the feasibility and accuracy of the proposed model in clinical practice, we freshly re-collected plantar pressure images of 46 participants from June 2022 to August 2023 as a new dataset, including 16 children and 30 adults, with the same inclusion and exclusion criteria as before **(** Supplementary Table S1 **)**. We inputted the datasets into different DL models and compared the obtained results with the original ML and clinical diagnosis results. The accuracy of FeetMapping^Ⓡ^ plantar pressure measurement system (NeuCognic, Jiangsu, China), ResNet-50-based multilabel classification algorithm, improved YOLO-v5 with SE or CBAM attention mechanism, and original YOLO-v5 were 0.630, 0.826, 0.652, 0.717, and 0.652, respectively (Figure 8).

## Discussion

In this study, we aimed to improve the YOLO-v5 DL model for foot classification based on plantar pressure images. We extracted middle foot features and incorporated SE and CBAM attention mechanisms. A comparison of the two modeling methods revealed that the modified YOLO-v5 model and the multilabel classification task greatly improved the performance of the flat foot classification system. Clinical verification showed that both models performed well, with the multilabel classification task yielding higher accuracy. Therefore, the ResNet-50-based multilabel classification algorithm is suitable for foot diagnosis and treatment prognosis.

This study highlighted the limitations of existing models in identifying soft tissue health conditions. Although the YOLO model excels in bone X-ray detection (Jiang et al., 2022; Li et al., 2023), it cannot identify soft tissue health issues. In this study, we used plantar pressure image data to conduct early screening for flat feet because they provide valuable information regarding lower limb dynamics and foot health.

The YOLO series is a one-stage target detection algorithm. YOLO-v1 divides the input image into a grid with uniform size; considers the prediction of the target and the target boundary box in the grid as a regression problem; and obtains the location, confidence, and category of the object (Redmon et al., 2016). However, YOLO-v1 poses challenges in accurately positioning objects and yields a low recall rate. Therefore, k-means clustering is incorporated in YOLO-v2, and the prior anchor frame is used for migration constraint and confidence prediction of target position; this further improves the performance of YOLO-v1 (Redmon and Farhadi, 2017). Nevertheless, during target detection, the regression parameters of the center position of the prior anchor frame in YOLO-v2 are not effectively constrained, which may cause the prediction anchor frame to appear in any position of the original image, resulting in low prediction accuracy. Furthermore, SoftMax activation classification is applicable only to a single target and not for multiple classifications. As such, logistic regression is incorporated in YOLO-v3 for parameters in the central location, limiting them to the range of 0–1, thereby greatly improving the detection accuracy. Furthermore, multiple independent logistic regression classifiers are used to replace SoftMax to improve the accuracy of multiobjective classification (Redmon and Farhadi, 2018). Based on YOLO-v3, YOLO-v4 incorporates advanced techniques such as Bag-of-Freebies (data enhancement, regularization, and loss function improvement) and Bag-of-Specials (enhancement of model sensitivity field, introduction of attention mechanism, feature integration, and post-processing method) methods to further improve detection efficiency (Bochkovskiy et al., 2020). The prior anchor frame of YOLO-v5 is similar to that of YOLO-v3 and YOLO-v4; however, YOLO-v5 incorporates the training prediction anchor frame into the network (Wang et al., 2022). During training, the optimal anchor frame value of different training sets is calculated adaptively, making the model applicable to various datasets and greatly improving the positioning accuracy for different tasks and datasets.

In this study, we also addressed the phenomenon of unilateral arch collapse observed in some flat feet patients as it can lead to asymmetrical force distribution and potential complications (Zhu et al., 2021; Kim and Lee, 2022). To address this issue, we adjusted the task strategy by focusing on the recognition of the foot arch and employed multilabel classification using ResNet-50, which yielded good results. Thermal maps generated using Grad-CAM revealed the importance of force distribution in the metatarsal region in addition to the midfoot. This is in agreement with previous studies (Szczepanowska-Wołowiec et al., 2021).

Common abnormal foot types include flat foot and high-arched foot. However, Buldt et al. (2018) observed no significant difference in plantar pressure between high arch feet and normal feet. Therefore, to avoid introducing bias to the model, we focused on the plantar pressure distribution of flat and normal feet in this study. Flat feet can lead to various health problems and affect daily functioning and quality of life (Chou et al., 2021; 2021; Moon and Jung, 2021). However, the latent early clinical manifestations of flat feet are often overlooked, and traditional diagnostic methods such as *X*-rays are less motivating for patients due to radiation and cost concerns. The use of plantar pressure measurement systems provides a simple screening method for flat feet but has limitations in terms of accuracy and sensitivity. In this study, we proposed a DL model and a multilabel classification algorithm as a novel and more accurate approach for foot type discrimination.

We observed differences in plantar pressure distribution between flat and healthy feet but no statistically significant difference in pressure distribution on the heel. This can be attributed to long-term compensatory or equilibrium mechanisms in the lower limbs of flat feet patients. The small number of participants included in this study posed challenges in model prediction, necessitating the need for further investigations to explore the relationship between these conditions and the subjects’ mental or physical health and to improve data collection methods.

## Limitations of the study

Limited datasets: In this study, data collected from a single healthcare facility by using specific equipment and evaluators were used. This limits the generalizability of the training model to datasets from different centers, devices, and evaluators. In addition, high arched feet were not included in this study, and a model suitable for high arched feet needs to be developed. To prevent overfitting and ensure robustness, future studies should include larger and more diverse datasets from multiple centers and devices.

## Conclusion

In this study, we clinically validated that the multilabel classification task and the improved YOLO-v5 model improve the performance of the plantar flat press system in foot classification. The multilabel classification task achieved higher accuracy. The pressure ratios of all plantar regions contribute to foot-type recognition, not limited to the arch of the foot. The multilabel classification algorithm based on ResNet-50 is suitable for foot diagnosis and treatment prognosis.

## Data Availability

The raw data supporting the conclusion of this article will be made available by the authors, without undue reservation.
