# Supplementary material for: A deep learning method for foot-type classification using plantar pressure images
Source: Front Bioeng Biotechnol. 2023 Sep 11;11:1239246. doi: 10.3389/fbioe.2023.1239246 (PMC10519788; doi:10.3389/fbioe.2023.1239246)
Supplement: Supplementary file 1 [file Table1.DOCX]

Supplementary Table 1. General information of prospective data sets

|  | **Healthy feet (n=16)** | **Flat feet (n=20)** | **one flat foot (n=10)** | ***P*** |
| --- | --- | --- | --- | --- |
| Female, n | 8 | 11 | 6 |  |
| Children, n^*^ | 4 | 9 | 3 |  |
| Age, y | 27.75±19.94 | 20.55±13.19 | 30.10±19.34 | 0.167 |
| Body mass（kg） | 58.44±14.40 | 52.63±14.52 | 61.40±19.33 | 0.302 |
| Body mass index（kg/m^2^） | 21.20±3.34 | 20.60±3.09 | 22.99±4.77 | 0.234 |

^*^Children: ≤18 years old
