# Supplementary figures and images for: A deep learning method for foot-type classification using plantar pressure images
Source: Front Bioeng Biotechnol. 2023 Sep 11;11:1239246. doi: 10.3389/fbioe.2023.1239246 (PMC10519788; doi:10.3389/fbioe.2023.1239246)

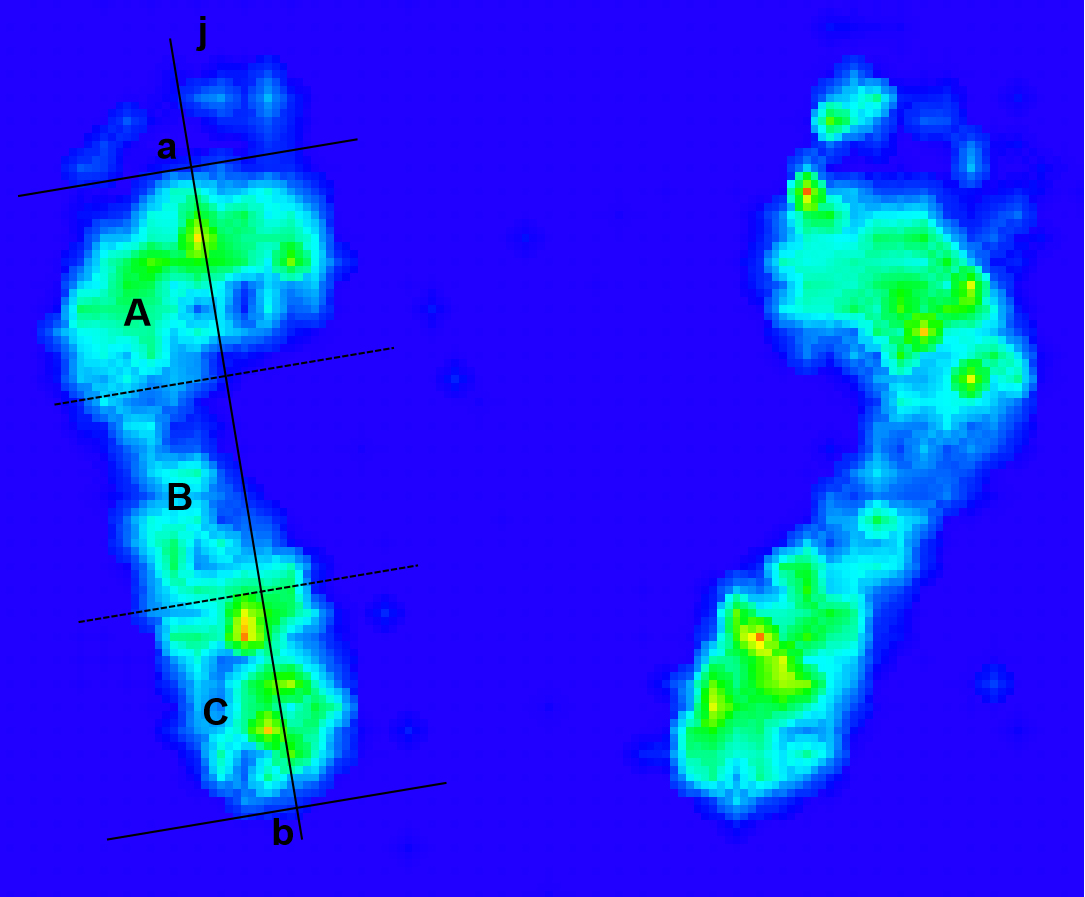

Supplement: Supplementary file 2 [file Image1.JPEG]

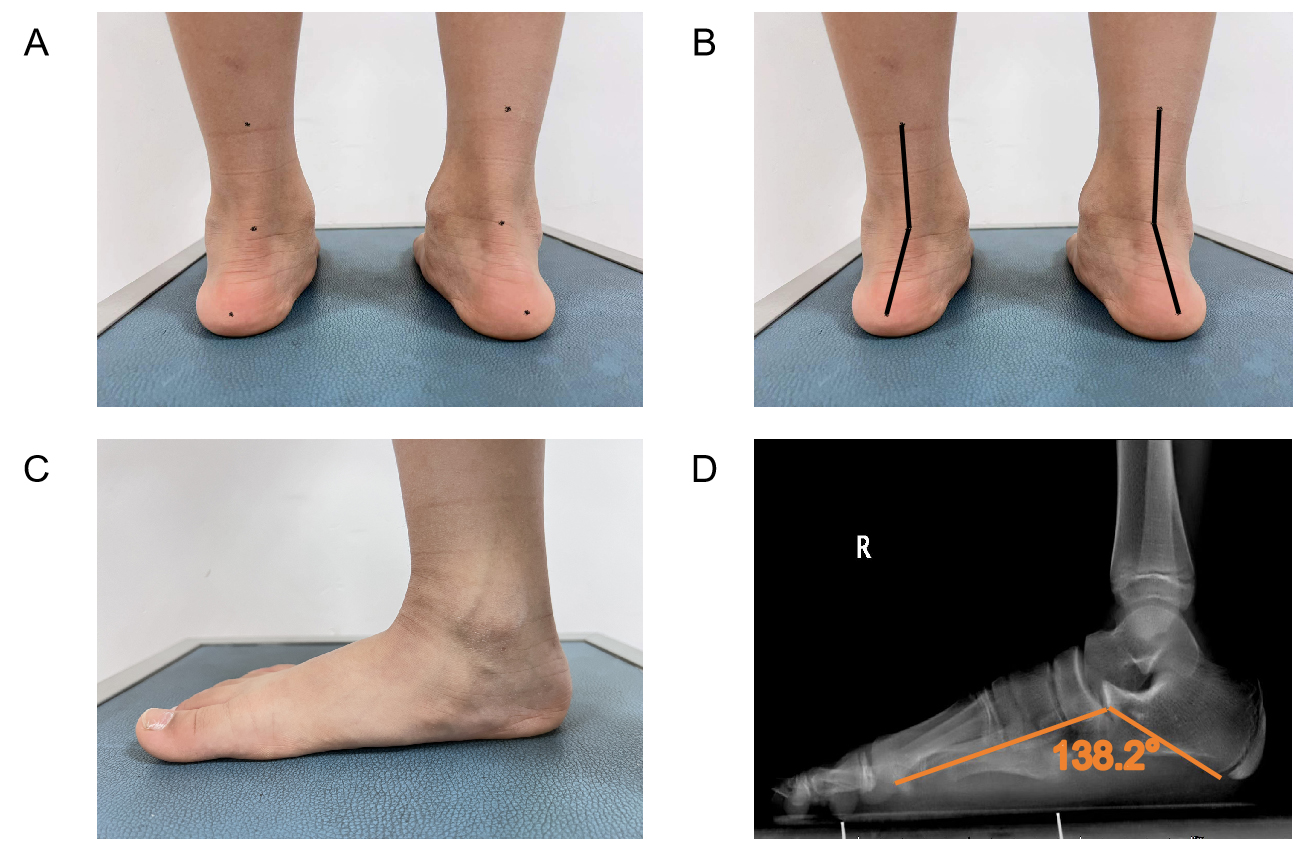

Supplement: Supplementary file 3 [file Image2.JPEG]
